# Mass-Specific Metabolic Rate Influences Sperm Performance through Energy Production in Mammals

**DOI:** 10.1371/journal.pone.0138185

**Published:** 2015-09-15

**Authors:** Maximiliano Tourmente, Eduardo R. S. Roldan

**Affiliations:** Reproductive Ecology and Biology Group, Museo Nacional de Ciencias Naturales (CSIC), Madrid, Spain; Clermont-Ferrand Univ., FRANCE

## Abstract

Mass-specific metabolic rate, the rate at which organisms consume energy per gram of body weight, is negatively associated with body size in metazoans. As a consequence, small species have higher cellular metabolic rates and are able to process resources at a faster rate than large species. Since mass-specific metabolic rate has been shown to constrain evolution of sperm traits, and most of the metabolic activity of sperm cells relates to ATP production for sperm motility, we hypothesized that mass-specific metabolic rate could influence sperm energetic metabolism at the cellular level if sperm cells maintain the metabolic rate of organisms that generate them. We compared data on sperm straight-line velocity, mass-specific metabolic rate, and sperm ATP content from 40 mammalian species and found that the mass-specific metabolic rate positively influences sperm swimming velocity by (a) an indirect effect of sperm as the result of an increased sperm length, and (b) a direct effect independent of sperm length. In addition, our analyses show that species with higher mass-specific metabolic rate have higher ATP content per sperm and higher concentration of ATP per μm of sperm length, which are positively associated with sperm velocity. In conclusion, our results suggest that species with high mass-specific metabolic rate have been able to evolve both long and fast sperm. Moreover, independently of its effect on the production of larger sperm, the mass-specific metabolic rate is able to influence sperm velocity by increasing sperm ATP content in mammals.

## Introduction

The basal metabolic rate represents the amount of energy transformation within an organism per time unit and constitutes perhaps the most fundamental biological parameter since it is statistically correlated with and functionally linked to numerous other traits [1–3]. Since Max Kleiber’s seminal work [4], the basal metabolic rate has been shown to scale with body mass as a decreasing allometric power law (exponent <1) in many metazoan groups [5]. There has been considerable debate about the precise value of the scaling exponent [1, 2], which ranges from ~2/3, based on surface-to-volume ratio [6, 7], to ~3/4, based on the properties of optimized resource distribution networks [8]. A recent study in mammals [9] has found that, while the exponent remains <1, its precise value is not constant for the analyzed body size range, responding to a quadratic curvature that increases with (log) body size. Associated with this relationship, there is the concept of “mass-specific metabolic rate”, which is defined as the ratio between the basal metabolic rate of an organism and its body mass (the rate at which an organism consumes energy per gram of body weight). In other words, it represents the “metabolic intensity” of a species [10]. By simple mathematical properties, this parameter is associated with body size by a negative power law in most eukaryote heterotrophic groups [5].

More importantly, the greatest portion of the basal metabolic rate of a whole organism is determined by the basal metabolic rate of tissues of its internal organs (minus skeletal muscle) [11], which in turn is a result of the basal metabolic rate of cells within these tissues [10, 12] and has been associated with the gradient-retention properties of cellular membranes [13]. As a result of the allometric scaling of the basal metabolic rate at the organism level, the increase in body size imposes variations in the metabolic properties of the cells. Recent evidence has revealed that, in mammals, most cell types follow a strategy in which the cellular metabolic rate depends on body size [14]. Thus, small species have higher cellular metabolic rates, which results in faster rates of processing energy and resources. This appears to be an intrinsic property of the tissue and is not due to differences in extracellular space or tissue protein content, which are relatively constant in all mammalian species examined [15]. Moreover, in mammals, numerous physiological processes at organ [16, 17] and tissue levels [15, 18], as well as the whole body turnover (synthesis *plus* degradation) rates of proteins [19], r-RNA, t-RNA, and m-RNA [20], have been reported to scale with body mass in a similar direction as the metabolic rate. On the other hand, the metabolic rate of cell lines from mammalian species varying in body mass are known to gradually gain independence from its parent organism’s body size along successive generations when cultured under *in vitro* conditions [21, 22]. In light of this evidence, it has been suggested that there is some form of constant stoichiometry related to overall metabolic activity that controls the metabolic rate of individual cells within tissues [10].

Recent studies have revealed that the mass-specific metabolic rate (measured at organism level) may act as a constraint for the length of spermatozoa [23, 24]. Thus, among eutherian and metatherian mammals, small species with high mass-specific metabolic rates are associated with long sperm while large species with low mass-specific metabolic rates have shorter sperm. This has led to the hypothesis that a low mass-specific metabolic rate would constrain the resource turnover rate of spermatogenic cells, which would prevent large species (with low mass-specific metabolic rates) from producing long sperm efficiently [23, 24]. However, the organism level mass-specific metabolic rate may affect the performance of sperm cells in an additional manner: sperm energetic metabolism may be constrained by their resource turnover rate at the cellular level if sperm cells retain the metabolic intensity of their parent organisms. In line with this idea, a comparative study found that sperm motility and viability increased with the relative metabolic rate, while morphological normality and acrosome integrity did not, suggesting that the mass-specific metabolic rate affects ejaculate traits linked to cellular energetics and maintenance of membrane integrity [25].

At the cellular level, mitochondria generate ATP to provide for different biosynthetic processes, maintenance of transmembrane ion gradients, and cell mechanical movement, which accounts for approximately 70% of the basal metabolic rate [26]. The availability of ATP is fundamental for a multitude of cellular processes that are key for fertilization in mammalian spermatozoa, such as capacitation [27, 28] and acrosome exocytosis [29]. Moreover, sustained motility, active protein phosphorylation, and ion regulation generate exceptionally high energetic demands in spermatozoa relative to other cell types [30, 31].

Mammalian spermatozoa swim forward as a consequence of flagellar movement, which is, in turn, the result of ATP hydrolysis by the dyneins (ATPases) associated with the axonemal microtubules. In mammals, sperm produce ATP by means of two main metabolic pathways: oxidative phosphorylation by mitochondria in the midpiece, or by glycolysis in the principal piece [32–34]. While the relative contribution of each metabolic pathway to total ATP content seems to vary among species and remains the topic of considerable debate [34–36], ATP consumption in processes related to motility represents a high fraction of the sperm energetic demands [37, 38]. Intraspecific studies show a close association between sperm internal ATP levels and sperm motility, flagellum beating frequency and swimming velocity [32, 34, 39]. More importantly, a recent comparative study in rodents revealed a positive relationship between ATP concentration in the sperm flagellum, and sperm swimming velocity and proportion of motile cells (40).

In the present study, we sought to evaluate the influence of the mass-specific metabolic rate in sperm swimming velocity by variation in sperm ATP content in mammals. Mammals are unique in that they show a vast range of body sizes with some species achieving the largest sizes of any living animal on earth. Thus, any size-dependent processes are likely to be particularly pronounced within this taxon. We hypothesized that the mass-specific metabolic rate of the parent organisms would be retained by their sperm cells, thus affecting their energy production capability, which in turn would impact on their swimming velocity. We predicted that sperm of species with high mass-specific metabolic rates would have higher ATP concentrations and swim at higher velocities than those from species with lower mass-specific metabolic rates.

## Materials and Methods

### Sperm parameters, relative testes size and mass-specific metabolic rate

Data were obtained from the literature for all variables analyzed. Data on body mass (g), testes mass (g), total sperm length (μm), and sperm straight-line velocity (μm s-^1^) from freshly collected, non-capacitated sperm were obtained for 40 eutherian mammal species (18 families) (S1 Table). Data on sperm ATP content (amol cell^-1^) were available for a subset of 22 species (S1 Table). To ensure data quality and comparability, data were from adult, healthy, reproductively-mature specimens. In the case of studies that evaluated sperm parameters on sperm collected from the cauda epididymis (all rodent species and *Macaca mulatta*), the values considered where those obtained after activation of sperm motility upon its dilution on culture medium. Sperm stored in the cauda epididymis are capable of full motility upon dilution and, after activation, the energetic metabolism of mammalian sperm is considered to be equivalent to that of ejaculated sperm [40, 41]. In cases in which samples under study were incubated, data were taken from measurements that were closest to sample collection. When data came from studies that evaluated cryopreservation (freezing/thawing) effects, we only used values of samples before freezing. In the case of data that came from experimental studies or clinical trials, only values of the control group were used. When possible, data were collected from studies in which the highest number of variables was analyzed. Regarding total sperm length, only species for which this parameter was measured from digital images were included in the dataset, excluding species for which the only data available were “approximations from Retzius’ illustrations” because they lacked a proper reference scale. Straight-line velocity (VSL) is the distance measured in a straight-line between the beginning and the end of the trajectory covered by a sperm cell in one second. We chose to use VSL as the velocity parameter since (a) this parameter is a commonly used measure of sperm swimming velocity and is significantly correlated with other sperm swimming parameters such as curvilinear velocity (VCL) or average-path velocity (VAP) in mammals [42, 43], (b) it shows less variability than VCL and VAP in response to differences in the settings of computer aided sperm analysis systems (especially when different frame-rates are used), and (c) VCL and VAP values were not available in all the studies from which data were collected.

Additionally, information on basal metabolic rate (ml O_2_ h^-1^) was available in the literature for 34 of the 40 species (S1 Table). When possible, we only included data that was measured in adult, resting, normothermic, post-absorptive, inactive and conscious specimens. However, these conditions can be difficult to achieve in mammals in which the digestive tract supports significant fermentation such as artiodactyls, macropods, or lagomorphs, or in small highly active species [44]. The mass-specific metabolic rate (ml O_2_ h^-1^ g^-1^) was calculated as the ratio between the basal metabolic rate and body mass. Since body mass measurements taken to calculate relative testes size usually belong to mature males and the basal metabolic rates usually represent a mean value for the species, we used additional body mass measurements (BMASS2 in S1 Table) from the same sources of the basal metabolic rate values to calculate the mass-specific metabolic rate.

In those cases in which different values for the same variable and species were available from different studies, averages were used to obtain a representative measure. Since not all data were available for each species, the number of species used for each analysis is indicated.

### Data analysis

To assess the effect of the mass-specific metabolic rate on sperm straight-line velocity and total sperm length, simple linear regressions were performed using both sperm traits as dependent variables and the mass-specific metabolic rate as predictor. Moreover, since sperm length has been reported to be positively associated with sperm velocity in mammals [45], we tested this relationship by means of a simple linear regression using sperm straight-line velocity as dependent variable and total sperm length as predictor. Since this relationship was confirmed in our dataset (PGLS: slope = 0.64, *p*<0.0001, *R* ^*2*^ = 0.35, S2 Fig), we used a multiple regression model to test the effect of the mass-specific metabolic rate on sperm straight-line velocity while controlling for sperm length by including both the mass-specific metabolic rate and total sperm length as predictors. Partial residuals were estimated from the multiple regression analysis in order to graphically represent the association between mass-specific metabolic rate and sperm straight-line velocity, while extracting the effect of total sperm length in the statistical model. A partial regression plot was constructed by estimating the residuals of a regression between sperm straight-line velocity and total sperm length, and plotting them against the residuals from a regression between mass-specific metabolic rate and total sperm length.

Additionally, we tested for the effect of the mass-specific metabolic rate on the ATP amount per sperm. Since larger cells might contain greater quantities of ATP due to increased internal volume, differences in cell size should be taken into account when testing for possible differences in sperm ATP concentration. Since data on sperm volume is scarce and relatively difficult to obtain for mammals [46, 47], and in cylindrically-shaped objects volume is proportional to length, we calculated the “length-adjusted ATP concentration” (amol μm^-1^) as the ratio between the amount of ATP per sperm for each species and its total sperm length [48]. Thus, we performed simple linear regression using ATP amount per sperm and length-adjusted ATP concentration as dependent variables and the mass-specific metabolic rate as predictor in order to assess the effect of the mass-specific metabolic rate on the absolute and length-proportional amount of ATP per sperm. Moreover, both ATP related variables were used as predictors of sperm straight-line velocity in separate simple linear regressions to evaluate the effect of ATP content on sperm velocity.

Finally, the trends reported by previous studies regarding the effect of sperm competition on the mass-specific metabolic rate, sperm length and sperm velocity [23, 24, 45, 48] were examined by multiple linear regressions using sperm traits and the mass-specific metabolic rate as dependent variables and body mass and testes mass as predictors [49].

In order to avoid the effect of spurious correlations caused by phylogenetic association between closely related species rather than selective evolution [50, 51], all regressions were carried out using phylogenetic generalized least-squares (PGLS) analyses [52]. PGLS includes the phylogenetic structure of a given topology within a standard linear model as a covariance matrix assuming a predetermined evolutionary model, and estimates (via maximum likelihood) a phylogenetic scaling parameter lambda (*λ*) of the tree’s branch lengths that fits evolution by Brownian motion. Lambda values close to 0, indicate that the relationship between the variables is highly independent of phylogeny, whereas lambda values close to 1 indicate a strong association of the variables with phylogeny. Additionally, we calculated the effect size *r* obtained from the models *t*-values [53]; effect sizes > 0.5 were considered as large [54]. Non-central confidence limits (CLs) for *r*, which indicate statistical significance if 0 is not contained within the interval [55], were also calculated.

Statistical analyses were performed using the CAPER v0.5 [56] package for R (v3.0.1; R Foundation for Statistical Computing 2013) with *P* < 0.05 regarded as statistically significant All variables were log_10_ transformed prior to analysis. Since a complete phylogeny for all species analysed was not available, a phylogenetic reconstruction was used to perform the PGLS (S2 Fig). This reconstruction was based on Bininda-Emonds et al. (2007) for the determination of the phylogenetic position of the higher groups (orders and families). Other phylogenies were used to resolve within-group relationships, namely Prothero & Foss (2007) and Agnarsson & May-Collado (2008) for Artiodactyla; Flynn *et al*. (2005) for Carnivora; Robinson & Matthee (2005) for Lagomorpha; Poux & Douzery (2004), Xing et al. (2005) and Baena *et al*. (2007) for Primates; and Fabre *et al*. (2012) for Rodentia.

## Results

**Fig 1:**
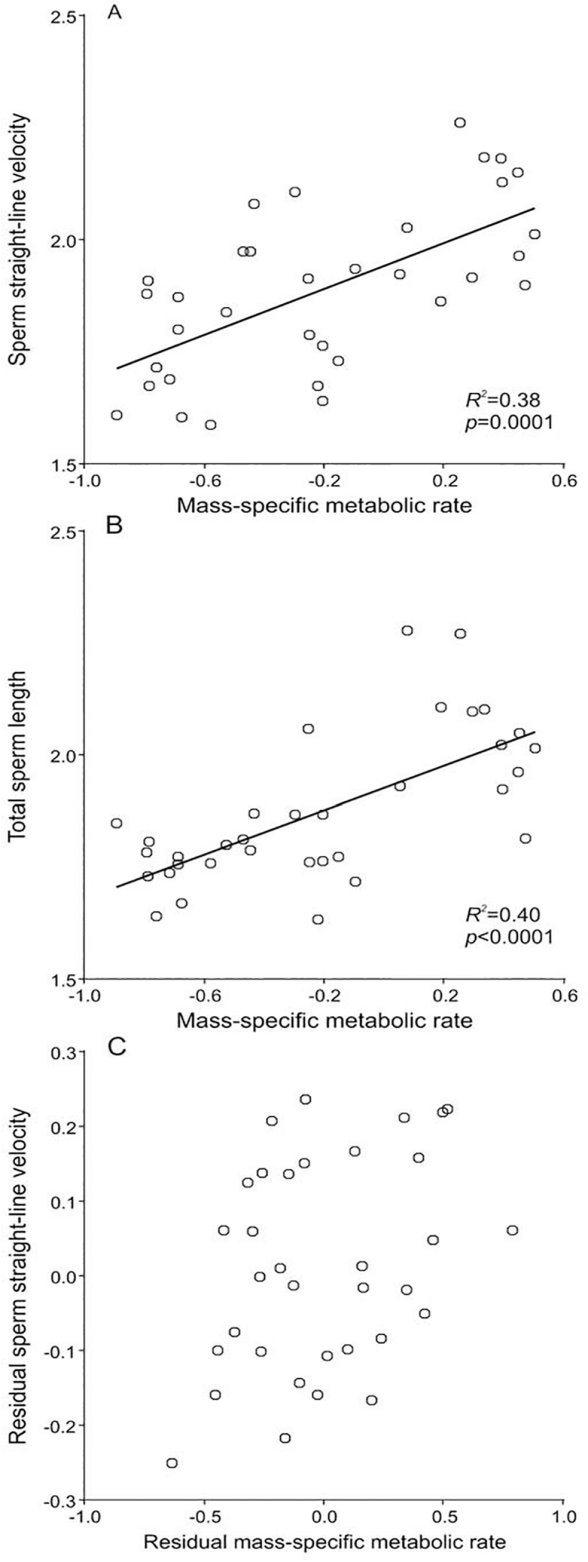
Mass-specific metabolic rate and sperm parameters in eutherian mammals. Relationships between mass-specific metabolic rate (ml O_2_ h^-1^ g^-1^) and (*A*) sperm straight-line velocity (μm s^-1^), (*B*) total sperm length (μm), and (*C*) sperm straight-line velocity (μm s^-1^) after controlling for the effect of total sperm length (μm), in mammalian species. All variables were log_10_-transformed. Figure points in (*A*) and (*B*) represent data values. Figure points in (*C*) are partial residuals estimated from a regression between sperm straight-line velocity and total sperm length (Y-axis), and from a regression between mass-specific metabolic rate and total sperm length (X-axis). *R* ^*2*^ and *p*-values were estimated using phylogenetically controlled regression analyses (PGLS).

Sperm straight-line velocity showed a range of values from 38.6 μm s^-1^ in the chimpanzee (*Pan troglodytes*) to 182 μm s^-1^ in the golden hamster (*Mesocricetus auratus*) (S1 Table). This results in an estimated 4.7-fold increase in this parameter for the range of species examined. Sperm straight-line velocity showed a significant positive association with mass-specific metabolic rate (PGLS, slope = 0.06, *p* = 0.0001, *R* ^*2*^ = 0.38) (Fig 1A, Table 1).

**Table 1:** Relations between sperm length, sperm straight-line velocity and mass-specific metabolic rate in eutherian mammals.

Dependent variable	Independent variable	Slope	*R* ^*2*^	*p-*value	*t*-value	*λ* value	Effect size	CL(-)	CL(+)	n
Sperm straight-line velocity	Mass-specific metabolic rate	0.0592	0.38	**0.0001**	4.4282	<0.001^ns,*^	0.6164	**0.3672**	**1.0712**	34
Total sperm length	Mass-specific metabolic rate	0.2399	0.40	**<0.0001**	4.6637	<0.001^ns,*^	0.6304	**0.3900**	**1.0941**	34
Sperm straight-line velocity	Total sperm length	0.4783	0.49	**0.0144**	2.5924	<0.001^ns,*^	0.4221	**0.0982**	**0.8023**	34
	Mass-specific metabolic rate	0.1473		**0.0442**	2.098		0.3526	**0.0164**	**0.7204**	

Phylogenetically controlled multiple regression analyses (PGLS). Superscripts following the *λ* value indicate significance levels (n.s. *p*>0.05; *p<0.05) in likelihood ratio tests against models with *λ* = 0 (first position) and *λ* = 1 (second position). Effect size *r* calculated from the *t* values and the non-central 95% confidence limits (CLs) for the *z*-transformed value of *r* are presented. Confidence intervals excluding 0 indicate statistically significant relationships. *P*-values and CL that indicate statistical significance are shown in bold. All variables were log_10_-transformed. n: number of species.

Total sperm length exhibited a similar degree of variation, ranging from 42.9 μm in the Damaraland mole-rat (*Fukomys damarensis*) to 189.4 μm in the golden hamster (*Mesocricetus auratus*), with an estimated 4.4-fold increase throughout the range of variation. Sperm length was significantly related to mass-specific metabolic rate (PGLS, slope = 0.24, *p*<0.0001, *R* ^*2*^ = 0.40) (Fig 1B, Table 1).

Taken together, these results suggest that species with high mass-specific metabolic rate have been able to evolve both long and fast sperm. However, since sperm straight-line velocity correlates with total sperm length, as seen in a previous study [45] and confirmed here (see Materials and methods and S2 Fig), we tested the effect of the mass-specific metabolic rate on sperm velocity adding total sperm length as a covariate. The result of such analysis indicated that the significant positive relationship between sperm straight-line velocity and the mass-specific metabolic rate was maintained after controlling for the effect of total sperm length (Fig 1C, Table 1). This would suggest that, while an increase in mass-specific metabolic rate may have an indirect effect of sperm straight-line velocity as the result of an increased sperm length, it also has a positive and direct effect on sperm straight-line velocity, independently of sperm length.

Notably, mass-specific metabolic rate exhibited a wider range of variation than sperm straight-line velocity and total sperm length, ranging from 0.13 in the brown bear (*Ursus arctos*) to 3.2 in the steppe mouse (*Mus spicilegus*), which represents a 24.6-fold increase from the lower to the upper end of the range. Because differences in the mass-specific metabolic rate influence the kinetics of numerous cellular processes, including energy production in the form of ATP [10, 12], and ATP is necessary for flagellar beating, we first tested if there is a relationship between the mass-specific metabolic rate and the ATP amount per sperm, and then if the latter was related to sperm straight-line velocity.

**Fig 2:**
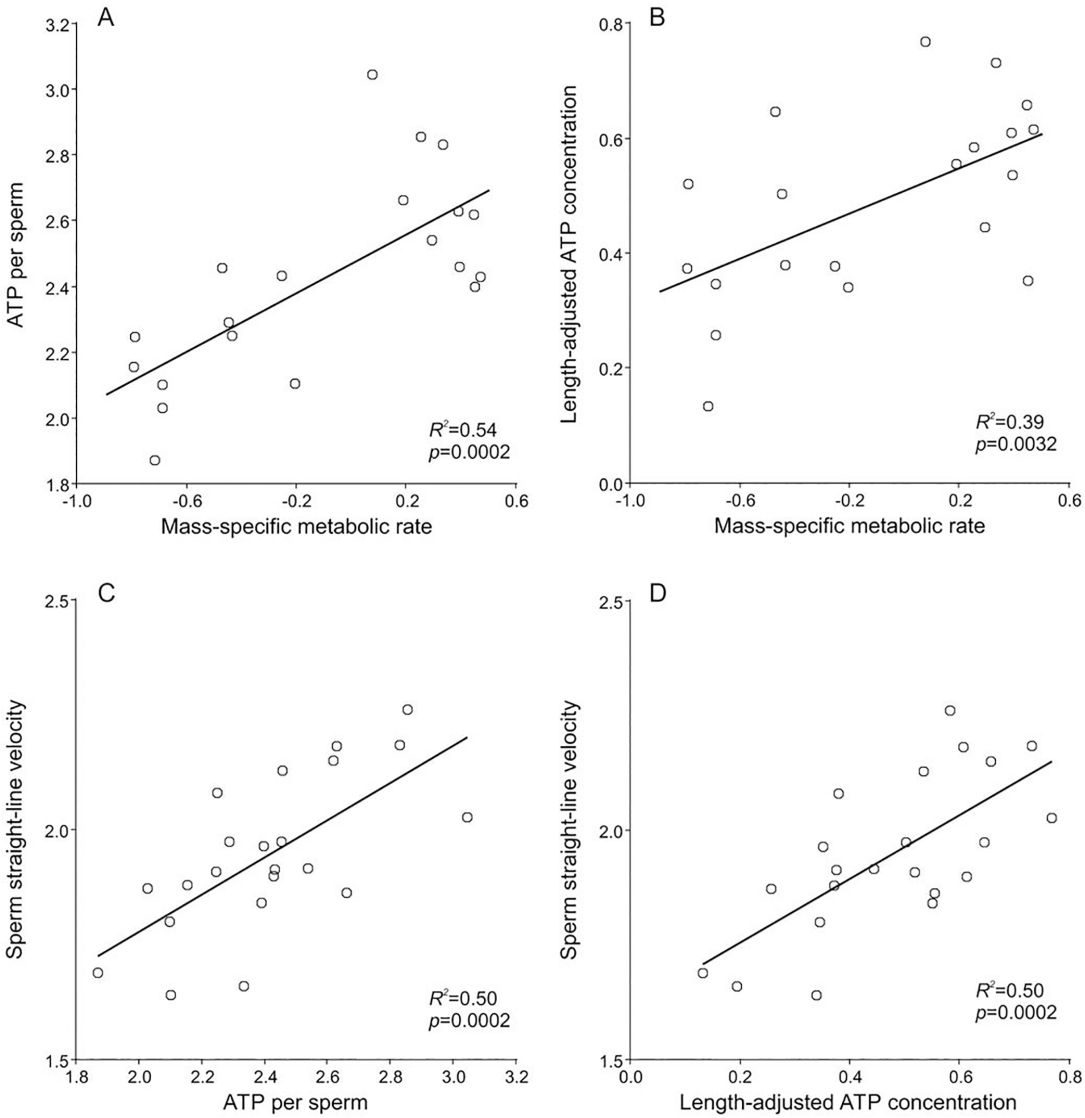
Mass-specific metabolic rate, sperm ATP content and sperm straight-line velocity in eutherian mammals. Relationships between (*A*) Mass specific metabolic rate (ml O_2_ h^-1^ g^-1^) and ATP amount per sperm (amol cell^-1^), (*B*) mass specific metabolic rate and length-adjusted ATP concentration (amol μm^-1^), (*C*) sperm straight-line velocity (μm s^-1^) and ATP amount per sperm, and (*D*) sperm straight-line velocity and length-adjusted ATP concentration, in mammalian species. All variables are log_10_-transformed. *R* ^*2*^ and *p*-values were estimated using phylogenetically controlled regression analyses (PGLS).

The estimated content of ATP per sperm cell ranged from 74.2 amol cell^-1^ in the boar (*Sus scrofa*) to 1107.5 amol cell^-1^ in the brown rat (*Rattus norvegicus*), which represents a 14.9-fold increase throughout the range of ATP values. We found that the ATP amount per sperm presented a significant positive association with the mass-specific metabolic rate (PGLS, slope = 0.49, *p* = 0.0002, *R* ^*2*^ = 0.54) (Fig 2A, Table 2). In addition, ATP amount per sperm was found to be positively correlated with total sperm length (PGLS, slope = 1.46, *p*<0.0001, *R* ^*2*^ = 0.74) (Table 2).

**Table 2:** Relations among sperm ATP amount, sperm length, sperm straight-line velocity and mass-specific metabolic rate eutherian in mammals.

Dependent variable	Independent variable	Slope	*R* ^*2*^	*p-*value	*t*-value	*λ* value	Effect size	CL(-)	CL(+)	n
ATP amount per sperm	Mass-specific metabolic rate	0.4851	0.54	**0.0002**	4.5606	<0.001^ns,*^	0.7322	**0.4580**	**1.4088**	20
ATP amount per sperm	Total sperm length	1.4562	0.74	**<0.0001**	7.6450	<0.001^ns,*^	0.8632	**0.8560**	**1.7553**	22
Length-adjusted ATP concentration	Mass-specific metabolic rate	0.2255	0.39	**0.0032**	3.4049	<0.001^ns,*^	0.6259	**0.2593**	**1.2100**	20
Sperm straight-line velocity	ATP amount per sperm	0.4113	0.50	**0.0002**	4.4722	<0.001^ns,*^	0.7071	**0.4317**	**1.3310**	22
Sperm straight-line velocity	Length-adjusted ATP concentration	0.7215	0.50	**0.0002**	4.5151	<0.001^ns,*^	0.7105	**0.4385**	**1.3378**	22

Phylogenetically controlled multiple regression analyses (PGLS). Superscripts following the *λ* value indicate significance levels (n.s. *p*>0.05; *p<0.05) in likelihood ratio tests against models with *λ* = 0 (first position) and *λ* = 1 (second position). Effect size *r* calculated from the *t* values and the non-central 95% confidence limits (CLs) for the *z*-transformed value of *r* are presented. Confidence intervals excluding 0 indicate statistically significant relationships. *P*-values and CL that indicate statistical significance are shown in bold. All variables were log_10_-transformed. n: number of species.

We also tested for a possible relationship between length-adjusted ATP concentration (a measure of ATP content corrected by total sperm length) and the mass-specific metabolic rate. Length-adjusted ATP concentration exhibited an increase throughout its range (4.3-fold) that was similar to the increases exhibited by sperm straight-swimming velocity and total sperm length. The species representing the extremes of such range were, as in the case of sperm ATP content, the boar (1.36 amol μm^-1^), and the brown rat (5.85 amol μm^-1^). Length-adjusted ATP concentration also exhibited a significantly positive relationship with mass-specific metabolic rate (PGLS, slope = 0.23, *p* = 0.0032, *R* ^*2*^ = 0.39) (Fig 2B, Table 2). Overall, these results suggest that a higher mass-specific metabolic rate (a) could promote an increase of the amount of ATP per sperm, and (b) has a positive effect on the concentration of ATP per μm of sperm length.

Finally, we tested for associations between ATP levels and sperm velocity. Results revealed that sperm straight-line velocity has a significant positive relationship with ATP amount per sperm (PGLS, slope = 0.41, *p* = 0.0002, *R* ^*2*^ = 0.50) (Fig 2C, Table 2). Furthermore, this relationship was positive and significant when length-adjusted ATP concentration was used as a predictor of sperm velocity (PGLS, slope = 0.72, *p* = 0.0002, *R* ^*2*^ = 0.50) (Fig 2D, Table 2). These results indicate that in order to achieve high straight-line velocities mammalian sperm would require high amounts of ATP per cell, and high concentrations of ATP per unit of sperm cell length.

## Discussion

We found that sperm straight-line velocity was positively associated with the mass-specific metabolic rate after controlling for the effect of sperm length (a known predictor of sperm straight-line velocity in mammals; [45]). Previous studies in mammals [23, 24] have revealed that the mass-specific metabolic rate imposes an energetic constraint to the evolution of sperm length, presumably through limitations on the rate at which spermatogenic cells can process energetic resources to produce competitively long sperm [57, 58]. Thus, species that have low mass-specific metabolic rate tend to produce short sperm. Additionally, the length of sperm flagellum is positively associated with sperm straight-line velocity in mammals as a result of an increase in sperm propulsive force [45]. More importantly, the results in the present study indicate that the mass-specific metabolic rate influences sperm straight-line velocity independently of its effect on sperm size.

Our analyses show that sperm straight-line velocity is also positively related to ATP content per sperm. In mammals, sperm forward motility depends on the propulsive force generated by the beating of the flagellum, which is attained through ATP hydrolysis by dyneins associated with its microtubules [32–34]. Moreover, our results indicate that the association between sperm ATP content and sperm straight-line velocity remains significant when using length-adjusted ATP concentration as predictor, suggesting that the increase in sperm straight-line velocity is not only a cell-size related phenomenon, but that it also depends on the amount of ATP per length unit of the sperm flagellum.

Sperm velocity has been reported to be linked to sperm ATP content and concentration in muroid rodents, in which increased sperm ATP content seems to be promoted by an increase of the sperm competition level [48]. A previous study on the influence of metabolic rate on sperm quality in mammals found a positive association the between mass-specific metabolic rate and relative testes size [25]. However, other studies using broader datasets have failed to find any relation between these parameters [23, 24]. Furthermore, neither the mass-specific metabolic rate nor ATP content per cell were significantly related to relative testes mass in the present study (S2 Table).

Since the “metabolic intensity” of a whole organism (its mass-specific metabolic rate) can be regarded as the sum of the metabolic rates of its constituent cells divided by its total mass [10], the mass-specific metabolic rate is directly related to the cellular metabolic rate [21]. More importantly, this association may be interpreted in the opposite way: a measure of the mass-specific metabolic rate of a given organism would constitute an accurate approximation of the metabolic rate of its cells. Consistent with our hypothesis, our results show that ATP content per sperm is positively correlated with the mass-specific metabolic rate in mammals. The fact that this relationship retained its significance when using length-adjusted ATP concentration indicates that an increase in the mass-specific metabolic rate promotes an increase in the amount of ATP produced per length unit of the sperm flagellum. There is ample evidence that the rate of numerous processes that contribute to metabolic activity at the cellular and subcellular level vary accordingly to the mass-specific metabolic rate in mammals. Comparative studies in mammals have shown that the hepatocyte respiration rate [59], Na^+^-K^+^-ATPase molecular activity [13], and microsomal [13] and mitochondrial [59] lipid unsaturation reflect differences in the mass-specific metabolic rate. Moreover, a positive association between the O_2_ consumption rate to support mitochondrial ATP production and the mass-specific metabolic rate has been found [10, 12]. In this context, our results suggest that mammalian species with high mass-specific metabolic rates would produce sperm with the ability to transform resources into ATP at a faster rate, thus increasing the ATP concentration in the flagellum.

Two additional factors must be taken in account to fully understand the relationship between the mass-specific metabolic rate and energy production. Firstly, in order to meet the high ATP demands imposed by sperm motility, mammalian sperm use a broad array of metabolic pathways. These may involve oxidative phosphorylation, aerobic and anaerobic glycolysis, amino acid- and fatty acid-oxidation, and high-energy phosphagen hydrolysis [32–34, 60]. However, in mammalian somatic cells, the activity of enzymes involved in anaerobic glycolysis and those involved in aerobic metabolism show apparent contradictions in their responses to differences in mass-specific metabolic rate. Thus, while glycolytic enzymes tend to show an increased activity in species with low mass-specific metabolic rate [61, 62], enzymes involved in oxidative phosphorylation exhibit a positive association with mass-specific metabolic rate [10, 13]. These contrasting results may be explained by the fact that comparative analyses of the mammalian glycolytic rates are based on the activity of the lactate dehydrogenase (LDH) of the hind limb skeletal muscles [61, 62], whose metabolism is predominantly related to active locomotion and the maximal metabolic rate [63]. On the other hand, the basal metabolic rate (the parameter from which the mass-specific metabolic rate is estimated) represents the resting metabolism of an organism and its value is related to the activity of the internal organs, rather than of skeletal muscle, which results in a totally different allometric variation. Furthermore, glycolysis in mammalian spermatozoa uses a sperm-specific variant of LDH (LDH-C4) which, along with other enzymes of the glycolytic pathway, is bound to the fibrous sheath of the flagellum [64–66]. Thus, because of differences in the association with cellular structures and discrepancies in enzyme variants, comparisons between the activities of glycolytic enzymes present in muscle and spermatozoa may be particularly inaccurate and should be performed with caution.

Secondly, it could be argued that sperm perform their function in a fluid environment outside the organism that produced them, thus being isolated from the metabolic environment of their germinal tissue. In this regard, comparative studies have found that in mammalian tumoral cell [21] and fibroblast [22] lines cultured *in vitro*, the cellular metabolic rate does not scale with body size, and therefore with the whole organism mass-specific metabolic rate. Nevertheless, there are fundamental differences between sperm cells and somatic cell models: the body-size independent metabolic rate of *in vitro* cultured somatic cell lines seems to be the result of progressive modification in the number of mitochondria per cell along successive generations, apparently in response to O_2_ concentrations in the culture medium. On the other hand, mature mammalian sperm are unable to modify their number of mitochondria, and even less to produce successive cell generations.

In conclusion our results show that, independently of allowing for the production of larger sperm, the mass-specific metabolic rate is able to influence sperm velocity by increasing sperm ATP content in mammals. Since the mass-specific metabolic rate at the organism level defines the rate of metabolism at the cellular level, mature spermatozoa would retain the metabolic rate of the germinal cells from which they are produced, which would impact on their rate of conversion of resources to energy (ATP) destined to flagellar motility. These findings have important implications for the understanding of evolutionary forces that influence sperm form and function. Although there is ample evidence showing that several sperm traits evolve in response to specific selective pressures (i.e. postcopulatory sexual selection), more pervasive evolutionary forces, such as the scaling between the organism body size and cellular metabolism, may constrain changes in such traits.

## Supporting Information

S1 Fig Sperm straight-line velocity and sperm length in eutherian mammals. Relationships between sperm straight-line velocity (μm s^-1^) and total sperm length (μm), in eutherian mammals. All variables are log_10_-transformed.
(PDF)

S2 Fig Phylogenetic reconstruction for the 40 mammalian species utilized in the PGLS analysis. (PDF)

S1 Table Sperm length, sperm velocity, sperm ATP content, body size, testes size and basal metabolic rate in eutherian mammals. Abreviations: VSL: straight-line velocity (μm s^-1^). TSL: total sperm length (μm). BMASS: body mass (g). TMASS: testes mass (g). BMASS2: body mass (g) (this value is used to calculate mass-specific metabolic rate). BMR: basal metabolic rate (mlO_2_ h^-1^). ATP: amount of ATP per sperm (amol cell^-1^).
(PDF)

S2 Table Relations among mass-specific metabolic rate, ATP amount, and relative testes size in eutherian mammals. Phylogenetically controlled multiple regression analyses (PGLS). Superscripts following the *λ* value indicate significance levels (n.s. *p*>0.05; *p<0.05) in likelihood ratio tests against models with λ = 0 (first position) and *λ* = 1 (second position). Effect size *r* calculated from the *t* values and the non-central 95% confidence limits (CLs) for the *z*-transformed value of *r* are presented. Confidence intervals excluding 0 indicate statistically significant relationships. *P*-values and CL that indicate statistical significance are shown in bold. All variables were log_10_ transformed. n: number of species.
(PDF)
