# Supplementary figures and images for: Mass-Specific Metabolic Rate Influences Sperm Performance through Energy Production in Mammals
Source: PLoS One. 2015 Sep 15;10(9):e0138185. doi: 10.1371/journal.pone.0138185 (PMC4570794; doi:10.1371/journal.pone.0138185)

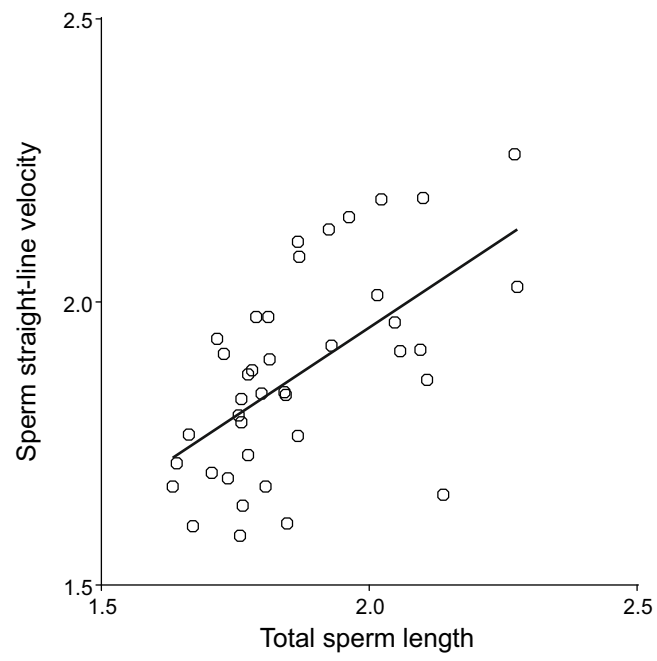

Supplement: S1 Fig — Relationships between sperm straight-line velocity (μm s-1) and total sperm length (μm), in eutherian mammals. All variables are log10-transformed. (PDF) [file pone.0138185.s001.pdf]

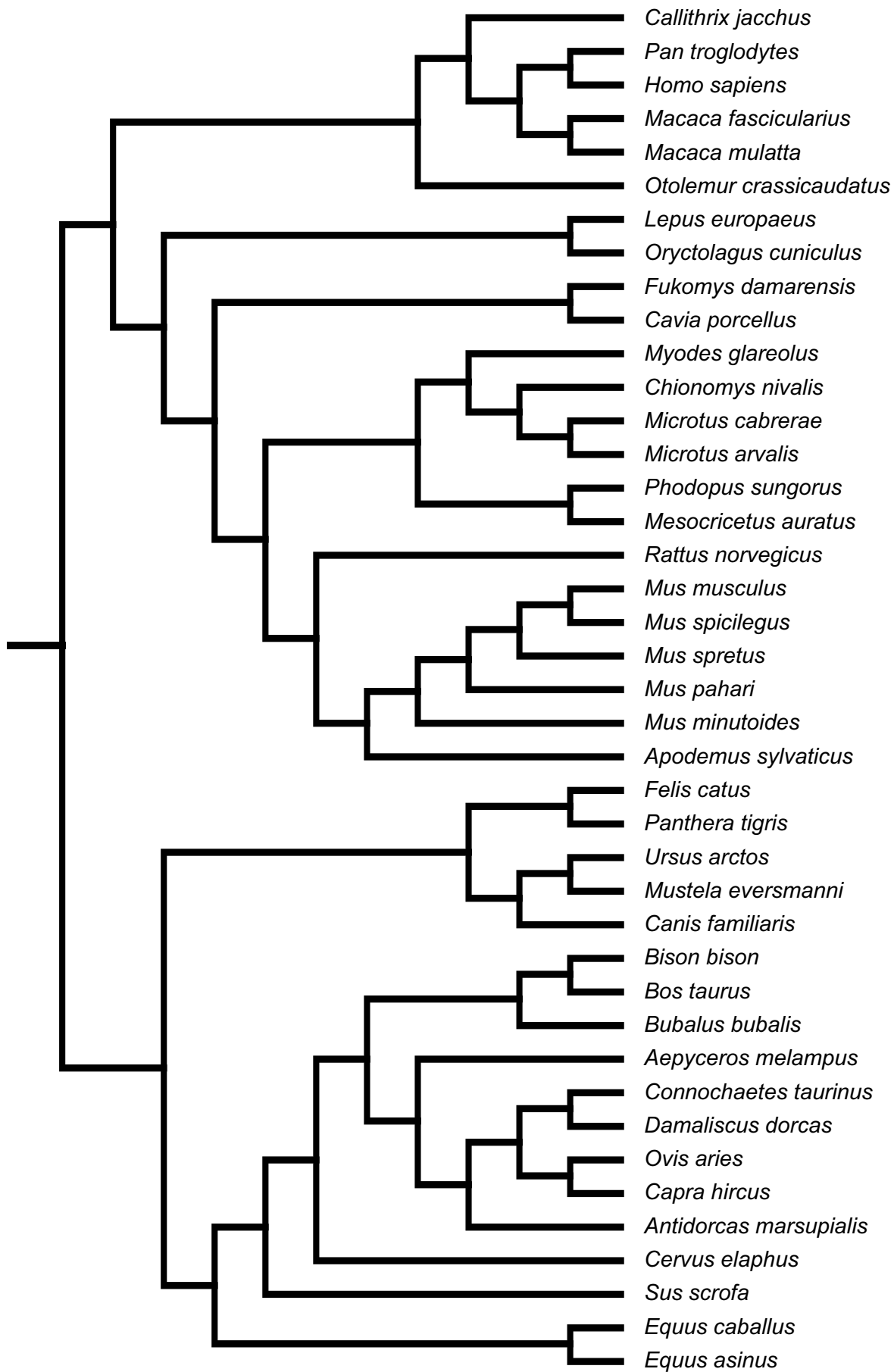

Supplement: S2 Fig — (PDF) [file pone.0138185.s002.pdf]
