# Supplementary material for: Mass-Specific Metabolic Rate Influences Sperm Performance through Energy Production in Mammals
Source: PLoS One. 2015 Sep 15;10(9):e0138185. doi: 10.1371/journal.pone.0138185 (PMC4570794; doi:10.1371/journal.pone.0138185)
Supplement: S1 Table — Abreviations: VSL: straight-line velocity (μm s-1). TSL: total sperm length (μm). BMASS: body mass (g). TMASS: testes mass (g). BMASS2: body mass (g) (this value is used to calculate mass-specific metabolic rate). BMR: basal metabolic rate (mlO2 h-1). ATP: amount of ATP per sperm (amol cell-1). (PDF) [file pone.0138185.s003.pdf]

S1 Table. Sperm length, sperm velocity, sperm ATP content, body size, testes size and basal metabolic rate in eutherian mammals.

Species	Family	Order	VSL	TSL	BMASS	TMASS	BMASS2	BMR	ATP	Ref. VSL	Ref. TSL	Ref. RTS	Ref. BMR	Ref. ATP
Aepyceros melampus	Bovidae	Artiodactyla	58.20	46.15	62500.00	120.000				1	42	52		
Antidorcas marsupialis	Bovidae	Artiodactyla	67.40	57.84	40500.00	71.000				1	43,44	53		
Bison bison	Bovidae	Artiodactyla	68.58	69.80	731400.00	523.600				2	2	54		
Bos taurus	Bovidae	Artiodactyla	81.10	53.53	680385.00	681.000	270000.00	44152.73	177.00	3,4	42	55	69,70	85,86
Bubalus bubalis	Bovidae	Artiodactyla	69.44	69.35	680000.00	652.000			246.10	5,6	45	56		87
Capra hircus	Bovidae	Artiodactyla	74.48	59.39	25420.00	156.800	32500.00	6698.80	107.23	7,8	42	57	70	8
Connochaetes taurinus	Bovidae	Artiodactyla	40.20	46.77	227000.00	306.000	168250.00	35511.15		9	42	52	70,71	
Damaliscus dorcas	Bovidae	Artiodactyla	49.80	50.82	72500.00	160.000				1	42	58		
Ovis aries	Bovidae	Artiodactyla	93.90	64.70	57172.73	222.991	30000.00	10200.00	285.50	10	42,46	53	72	85,88
Cervus elaphus	Cervidae	Artiodactyla	68.95	63.08	121800.00	217.000	67000.00	20012.54		11	47	55	73	
Sus scrofa	Suidae	Artiodactyla	48.85	54.60	39700.00	128.200	91250.00	17581.85	74.20	12,13	42,46	59	69,70	85,89
Canis familiaris	Canidae	Carnivora	93.90	61.40	21620.00	27.660	20800.00	7467.20	195.00	10,14	48,43	60	74	90
Felis catus	Felidae	Carnivora	53.58	59.35	3100.00	2.340	2617.00	1845.60		15,16	48,42	61	75	
Panthera tigris	Felidae	Carnivora	52.00	43.63	340500.00	24.400	137900.00	23994.60		17	42	53	76	
Mustela eversmanni	Mustelidae	Carnivora	58.10	73.73	1047.00	3.280	612.00	382.50		18	48,43	55	70	
Ursus arctos	Ursidae	Carnivora	40.51	70.35	180900.00	68.600	162428.57	20790.60		19	46,43	62	77	
Lepus europaeus	Leporidae	Lagomorpha	61.36	57.58	2950.00	14.552	3463.50	1948.15		20	49	63	75	
Oryctolagus cuniculus	Leporidae	Lagomorpha	43.65	58.02	2888.00	6.060	2204.93	1374.15	127.00	21,22,23	42	55	71,69,70	91
Equus asinus	Equidae	Perissodactyla	47.20	64.15	290000.00	202.340	177500.00	29287.50		24,25	42	53	78	
Equus caballus	Equidae	Perissodactyla	75.60	60.60	468000.00	416.000	387500.00	62473.00	143.00	26	42	55	74,79	85,92
Callithrix jacchus	Callithricidae	Primates	86.10	52.10	320.00	1.300	190.00	152.00		27	42	55	71	
Macaca fascicularis	Cercopitecidae	Primates	128.00	73.63	4787.00	35.700	4209.10	2118.79		28	42	55	75	
Macaca mulatta	Cercopitecidae	Primates	120.09	74.10	10430.00	76.000	6142.50	2256.10	177.33	29,30	42,43	55	77,70	93,94,95
Homo sapiens	Hominidae	Primates	63.05	56.90	63540.00	50.200	68825.00	14107.61	126.00	31,32	43	55	69,70	96,97
Pan troglodytes	Pongidae	Primates	38.60	57.36	44340.00	118.800	34150.00	9000.00		33	42	55	77	
Chionomys nivalis	Arvicolidae	Rodentia	151.35	105.23	46.99	0.940	32.80	81.02	426.76	34	34	38,64	78	38
Microtus arvalis	Arvicolidae	Rodentia	141.18	91.66	39.89	0.349	21.95	61.26	416.24	34	34	38,64	73,76	38
Microtus cabrerae	Arvicolidae	Rodentia	83.86	85.12	44.27	0.142	43.00	48.60		34	34	64	70	
Myodes glareolus	Arvicolidae	Rodentia	134.63	83.91	27.46	0.514	22.53	56.16	287.97	34	34	38,64	73,80,81,69	38
Fukomys damarensis	Bathyergidae	Rodentia	47.26	42.90	194.50	0.177	128.00	76.87		35	50	65	77	
Cavia porcellus	Caviidae	Rodentia	82.00	114.07	813.30	4.100	673.20	377.44	271.60	36	42,51	55	71,69,70	36,98
Mesocricetus auratus	Cricetidae	Rodentia	182.00	186.70	108.00	3.170	104.20	186.30	715.00	37	42	55	82,71,70	99
Phodopus sungorus	Cricetidae	Rodentia	72.75	127.99	46.39	1.004	30.47	47.03	459.39	38	38	38	71,69,70	38
Apodemus sylvaticus	Muridae	Rodentia	152.45	126.15	30.45	1.024	23.13	49.84	679.13	34	34	38,64	80,81,71,70	38
Mus minutoides	Muridae	Rodentia	79.32	65.22	4.91	0.097	8.08	23.93	267.99	38	38	38	70,83	38
Mus musculus	Muridae	Rodentia	82.21	124.64	23.30	0.139	23.26	45.74	346.53	34	34	38,64	78,69,77,70	38,100,101
Mus pahari	Muridae	Rodentia	45.63	137.60	32.23	0.127			215.49	38	38	38		38
Mus spicilegus	Muridae	Rodentia	102.74	103.54	14.49	0.409	14.71	47.07		34	34	64	84	
Mus spretus	Muridae	Rodentia	92.24	111.49	16.60	0.292	21.80	61.66	250.36	34	34	38,64	81,83	38
Rattus norvegicus	Muridae	Rodentia	106.47	189.40	379.63	3.058	226.81	271.62	1107.50	39,40,41	42	55,66,40,67,68	69,70	102,103

Abreviations: VSL: straight-line velocity (μm s^{-1}). TSL: total sperm length (μm). BMASS: body mass (g). TMASS: testes mass (g). BMASS2: body mass (g) (this value is used to calculate mass-specific metabolic rate). BMR: basal metabolic rate (mlO_2 h^{-1}). ATP: amount of ATP per sperm (amol $cell^{-1}$). RTS: references to the data for BMASS and TMASS (used to estimate relative testes size). MMR: references to the data for BMASS2 and BMR (used to estimate mass-specific metabolic rate).

References

1. Chatiza FP, Bartels P, Nedambale TL, Wagenaar GM. Computer assisted sperm analysis of motility patterns of postthawed epididymal spermatozoa of springbok (*Antidorcas marsupialis*), impala (*Aepyceros melampus*), and blesbok (*Damaliscus dorcas phillipsi*) incubated under conditions supporting domestic cattle in vitro fertilization. Theriogenology. 2012;78:402-14.
2. Pegge RB, Krishnakumar S, Whiteside D, Elkin B, Parlevliet JM, Thundathil JC. Sperm characteristics in plains (*Bison bison bison*) versus wood (*Bison bison athabascae*) bison. Theriogenology. 2011 Apr 15;75(7):1360-70.
3. Goovaerts IG, Hoflack GG, Van Soom A, Dewulf J, Nichi M, de Kruif A, et al. Evaluation of epididymal semen quality using the Hamilton-Thorne analyser indicates variation between the two caudae epididymides of the same bull. Theriogenology. 2006 Jul 15;66(2):323-30.
4. Muino R, Pena AI, Rodriguez A, Tamargo C, Hidalgo CO. Effects of cryopreservation on the motile sperm subpopulations in semen from Asturiana de los Valles bulls. Theriogenology. 2009 Oct 1;72(6):860-8.
5. Selvaraju S, Reddy IJ, Nandi S, Rao SB, Ravindra JP. Influence of IGF-I on buffalo (*Bubalus bubalis*) spermatozoa motility, membrane integrity, lipid peroxidation and fructose uptake in vitro. Anim Reprod Sci. 2009 Jul;113(1-4):60-70.
6. Koonjaenak S, Pongpeng P, Wirojwuthikul S, Johannisson A, Kunavongkrit A, Rodriguez-Martinez H. Seasonality affects post-thaw plasma membrane intactness and sperm velocities in spermatozoa from Thai AI swamp buffaloes (*Bubalus bubalis*). Theriogenology. 2007 Jun;67(9):1424-35.
7. Cox JF, Alfaro V, Montenegro V, Rodriguez-Martinez H. Computer-assisted analysis of sperm motion in goats and its relationship with sperm migration in cervical mucus. Theriogenology. 2006 Sep 1;66(4):860-7.
8. Berlinguer F, Madeddu M, Pasciu V, Succu S, Spezzigu A, Satta V, et al. Semen molecular and cellular features: these parameters can reliably predict subsequent ART outcome in a goat model. Reprod Biol Endocrinol. 2009;7:125.
9. Mata-Campuzano M, Álvarez M, Borragán S, Martínez-Pastor F, Nicolas M, Tamayo J, et al. Freezability of electroejaculated and epididymal spermatozoa from blue wildebeest (*Connochaetes taurinus*). Reprod Fertil Dev. 2008;21:179.
10. Holt WV, O'Brien J, Abaigar T. Applications and interpretation of computer-assisted sperm analyses and sperm sorting methods in assisted breeding and comparative research. Reprod Fertil Dev. 2007;19:709-18.
11. Malo AF, Garde JJ, Soler AJ, Garcia AJ, Gomendio M, Roldan ERS. Male fertility in natural populations of red deer is determined by sperm velocity and the proportion of normal spermatozoa. Biol Reprod. 2005 Apr;72(4):822-9.
12. Abaigar T, Holt WV, Harrison RAP, del Barrio G. Sperm subpopulations in boar (*Sus scrofa*) and gazelle (*Gazella dama mhorr*) semen as revealed by pattern analysis of computer assisted motility assessments. Biol Reprod. 1999;60:32-41.
13. Vyt P, Maes D, Rijsselaere T, Dejonckheere E, Castryck F, Van Soom A. Motility assessment of porcine spermatozoa: a comparison of methods. Reprod Domest Anim. 2004;39:447-53.
14. Rijsselaere T, Maes D, Hoflack G, de Kruif A, Van Soom A. Effect of body weight, age and breeding history on canine sperm quality parameters measured by the Hamilton-Thorne analyser. Reprod Domest Anim. 2007 Apr;42(2):143-8.
15. Stachecki JJ, Ginsburg KA, Leach RE, Randall Armant D. Computer-assisted semen analysis (CASA) of epididymal sperm from the domestic cat. J Androl. 1993;14(1):60-5.
16. Stachecki JJ, Ginsburg KA, Randall Armant D. Stimulation of cryopreserved epididymal spermatozoa of the domestic cat using the motility stimulants Caffeine, Pentoxyfiline, and 2'-Deoxyadenosine. J Androl. 1994;15(2):157-64.
17. Patil SB, Jayaprakash D, Shivaji S. Cryopreservation of semen of tigers and lions: Computerized analysis of the motility parameters of the spermatozoa. Curr Sci. 1998;75(9):930-5.
18. van der Horst G, Kitchin RM, van der Horst M, Atherton RW. The effect of the breeding season, cryopreservation and physiological extender on selected sperm and semen parameters of four ferret species: implications for captive breeding in the endangered black-footed ferret. Reprod Fertil Dev. 2009;21:351-63.
19. Nicolas M, Alvarez M, Gomes-Alves S, Mata-Campuzano M, Borragán S, Martinez-Pastor F, et al. Effects on brown bear (*Ursus arctos*) spermatozoa freezability of different extender and dilution ratios used for pre-freezing centrifugation. Eur J Wildlife Res. 2010;57(2):259-66.
20. Kozdrowski R. Assessment of the quality of European hare (*Lepus europaeus*, Pallas 1778) semen with conventional and computer methods. Bull Vet Inst Pulawy. 2007;51:569-73.
21. Castellini C, Lattaioli P, Bernardini M, Dal Bosco A. Effect of dietary a-tocopheryl acetate and ascorbic acid on rabbit semen stored at 5º C. Theriogenology. 2000;54:523-33.
22. Lavara R, Moce E, Lavara F, Viudes de Castro MP, Vicente JS. Do parameters of seminal quality correlate with the results of on-farm inseminations in rabbits? Theriogenology. 2005 Sep 15;64(5):1130-41.
23. Castellini C, Pizzi F, Theau-Clement M, Lattaioli P. Effect of different number of frozen spermatozoa inseminated on the reproductive performance of rabbit does. Theriogenology. 2006 Dec;66(9):2182-7.
24. Trimeche A, Renard P, le Lannou D, Barriere P, Tainturier D. Improvement of motility of post-thaw Poitou jackass sperm using glutamine. Theriogenology. 1996;45:1015-27.
25. Miro J, Lobo V, Quintero-Moreno A, Medrano A, Pena A, Rigau T. Sperm motility patterns and metabolism in Catalonian donkey semen. Theriogenology. 2005 Apr 1;63(6):1706-16.
26. Trimeche A, Yvon JM, Vidament M, Palmer E, Magistrini M. Effects of glutamine, proline, histidine, and betaine on post-thaw motility of stallion spermatozoa. Theriogenology. 1999;52:181-91.
27. Morrell JM. CASA as an aid to selecting sperm suspensions for artificial insemination in *Callithrix jacchus*. Int J Androl. 1997;20:287-96.
28. Mahony MC, Lanzendorf S, Gordon K, Hodgen GD. Effects of caffeine and dbcAMP on zona pellucida penetration by epididymal spermatozoa of cynomolgus monkeys (*Macaca fascicularis*). Mol Reprod Dev. 1996;43:530-5.
29. Baumber J, Meyers SA. Hyperactivated motility in rhesus macaque (*Macaca mulatta*) spermatozoa. J Androl. 2006 May-Jun;27(3):459-68.
30. Hung PH, Baumber J, Meyers SA, VandeVoort CA. Effects of environmental tobacco smoke in vitro on rhesus monkey sperm function. Reprod Toxicol. 2007 Jun;23(4):499-506.
31. Mortimer ST, Mortimer D. Kinematics of human spermatozoa incubated under capacitating conditions. J Androl. 1990;11(3):195-203.
32. Sloter E, Schmid TE, Marchetti F, Eskenazi B, Nath J, Wyrobek AJ. Quantitative effects of male age on sperm motion. Hum Reprod. 2006 Nov;21(11):2868-75.
33. Gould KG, Young LG. Functional parameters of chimpanzee (*Pan troglodytes*) sperm from ejaculates collected by rectal probe electrostimulation and by artificial vagina. Am J Primatol. 1996;39:115-22.
34. Gomez Montoto L, Varea Sanchez M, Tourmente M, Martin-Coello J, Luque-Larena JJ, Gomendio M, et al. Sperm competition differentially affects swimming velocity and size of spermatozoa from closely related muroid rodents: head first. Reproduction. 2011 Dec;142(6):819-30.
35. Maswanganye KA, Bennet NC, Brinders J, Cooney R. Oligospermia and azoospermia in non-reproductive male Damaraland mole-rats *Cryptomys damarensis* (Rodentia: Bathyergidae). J Zool. 1999;248(3):411-8.
36. Mújica A, Moreno-Rodríguez R, Naciff J, Neri L, Tash JS. Glucose regulation of guinea-pig sperm motility. J Reprod Fertil. 1991;92:75-87.
37. Shivaji S, Peedicayil J, Devi LG. Analysis of the motility parameters of in vitro hyperactivated hamster spermatozoa. Mol Reprod Dev. 1995;42:233-47.
38. Tourmente M, Rowe M, González-Barroso MM, Rial E, Gomendio M, Roldan ERS. Postcopulatory sexual selection increases ATP content in rodent spermatozoa. Evolution. 2013;67(6):1838-46.
39. Slott VL, Linder RE, Dyer CJ. Method of euthanasia does not affect sperm motility in the laboratory rat. Reprod Toxicol. 1994;8(4):371-4.
40. Kawaguchi TM, Kawachi M, Morikawa M, Kazuta H, Shibata K, Ishida M, et al. Key parameters of sperm motion in relation to male fertility in rats given a-chlorohydrin or nitrobenzene. J Toxicol Sci. 2004;29(3):217-31.
41. Immler S, Moore GD, Breed WG, Birkhead TR. By hook or by crook? morphometry, competition and cooperation in rodent sperm. PLoS One. 2007;2(1):e170.
42. Cummins JM, Woodall PF. On mammalian sperm dimensions. J Reprod Fertil. 1985;75:153-75.
43. Anderson MJ, Nyholt J, Dixson AF. Sperm competition and the evolution of sperm midpiece volume in mammals. J Zool. 2005;267(02):135.
44. Downing Meisner A, Klaus AV, O'Leary MA. Sperm head morphology in 36 species of artiodactylans, perissodactylans, and cetaceans (Mammalia). J Morphol. 2005 Feb;263(2):179-202.
45. Sharma AK, Gupta RC. Mensuration of spermatozoa from different levels of the reproductive tract of the buffalo-bull (*Bubalus bubalis*). Ann Biol Anim Bioch Biophys. 1978;18(3):717-20.
46. Gage MJG. Mammalian sperm morphometry. Proc R Soc Lond B Biol Sci. 1998;265:97-103.
47. Malo AF, Gomendio M, Garde J, Lang-Lenton B, Soler AJ, Roldan ERS. Sperm design and sperm function. Biol Lett. 2006 Jun 22;2(2):246-9.
48. Steklenev EP. Species characteristics of sperm morpholofy of individual members of the Order (Carnivora) in relation to hybridization. Tsitol Genet. 1975;9:142-6.
49. Kozdrowski R, Dubiel A, Dzmira S. Morphology of European brown hare (*Lepus europaeus*, Pallas 1778) semen. Bull Vet Inst Pulawy. 2006;50:383-5.

50. Maswanganye KA, Malherbe GP, Bennet NC. Ultrastructure of epididymal spermatozoa in three social African mole-rats of the genus *Cryptomys* (Rodentia: Bathyergidae): conservatism in sperm morphology. Afr Zool. 2005;40(2):293-300.
51. Gallardo MH, Mondaca FC, Ojeda RA, Köhler N, Garrido O. Morphological diversity in the sperms of caviomorph rodents. Mastozool Neotrop. 2002;9(2):159-70.
52. Gage MJ, Freckleton RP. Relative testis size and sperm morphometry across mammals: no evidence for an association between sperm competition and sperm length. Proc R Soc Lond B Biol Sci. 2003 Mar 22;270(1515):625-32.
53. Anderson MJ, Nyholt J, Dixson AF. Sperm competition affects the structure of the mammalian vas deferens. J Zool. 2004;264(1):97-103.
54. Helbig L, Woodbury MR, Haigh JC, Collins J, Barth AD. The seasonal fertility of North American bison (*Bison bison*) bulls. Anim Reprod Sci. 2007 Feb;97(3-4):265-77.
55. Kenagy GJ, Trombulak SC. Size and function of mammalian testes in relation to body size. J Mammal. 1986;67(1):1-22.
56. Pant HC, Sharma RK, Patel SH, Shukla HR, Mittal AK, Kasiraj R, et al. Testicular development and its relationship to semen production in Murrah buffalo bulls. Theriogenology. 2003;60(1):27-34.
57. Mekasha Y, Tegegne A, Abera A, Rodriguez-Martinez H. Body size and testicular traits of tropically-adapted bucks raised under extensive husbandry in Ethiopia. Reprod Domest Anim. 2008 Apr;43(2):196-206.
58. Cassinello J, Abaigar T, Gomendio M, Roldan ERS. Characteristics of the semen of three endangered species of gazelles (*Gazella dama mhorr*, *G. dorcas neglecta* and *G. cuvieri*). J Reprod Fertil. 1998;113:35-45.
59. Almeida FF, Leal MC, Franca LR. Testis morphometry, duration of spermatogenesis, and spermatogenic efficiency in the wild boar (*Sus scrofa scrofa*). Biol Reprod. 2006 Nov;75(5):792-9.
60. Woodall PF, Johnstone IP. Dimensions and allometry of testes, epididymides and spermatozoa in the domestic dog (*Canis familiaris*). J Reprod Fertil. 1988;82:603-9.
61. Franca LR, Godinho CL. Testis morphometry, seminiferous epithelium cycle length, and daily sperm production in domestic cats (*Felis catus*). Biol Reprod. 2003 May;68(5):1554-61.
62. White D, Berardinelli JG, Aune KE. Age variation in gross and histological characteristics of the testis and epididymis in grizzly bears. Ursus. 2005;16(2):190-7.
63. Tourmente M, Gomendio M, Roldan ERS. Sperm competition and the evolution of sperm design in mammals. BMC Evol Biol. 2011;11:12.
64. Gomez Montoto L, Magana C, Tourmente M, Martin-Coello J, Crespo C, Luque-Larena JJ, et al. Sperm competition, sperm numbers and sperm quality in muroid rodents. PLoS One. 2011;6(3):e18173.
65. Faulkes CG, Trowell SN, Jarvis JUM, Bennett NC. Investigation of numbers and motility of spermatozoa in reproductively active and socially suppressed males of two eusocial African mole-rats, the naked mole-rat (*Heterocephalus glaber*) and the Damaraland mole-rat (*Cryptomys damarensis*). J Reprod Fertil. 1994;100:411-6.
66. Slott VL, Suarez JD, Simmons JE, Perreault SD. Acute inhalation ixposure to epichlorohydrin decreases rat sperm velocity. Fundam Appl Toxicol. 1990;15:597-606.
67. Farombi EO, Adedara IA, Ebokaiwe AP, Teberen R, Ehwerhemuepha T. Nigerian Bonny light crude oil disrupts antioxidant systems in testes and sperm of rats. Arch Environ Contam Toxicol.
68. Wu PF, Chiang TA, Chen MT, Lee CP, Chen PH, Ko AM, et al. A characterization of the antioxidant enzyme activity and reproductive toxicity in male rats following sub-chronic exposure to areca nut extracts. J Hazard Mater. 2010 Jun 15;178(1-3):541-6.
69. Savage VM, Gillooly JF, Woodruff WH, West GB, Allen AP, Enquist BJ, et al. The predominance of quarter-power scaling in biology. Funct Ecol. 2004;18:257-82.
70. Sieg AE, O'Connor MP, McNair JN, Grant BW, Agosta SJ, Dunham AE. Mammalian metabolic allometry: do intraspecific variation, phylogeny, and regression models matter? Am Nat. 2009 Nov;174(5):720-33.
71. White CR, Seymour RS. Mammalian basal metabolic rate is proportional to body mass2/3. Proc Natl Acad Sci U S A. 2003 Apr 1;100(7):4046-9.
72. Elgar MA, Harvey PH. Basal metabolic rates in mammals: allometry, phylogeny and ecology. Funct Ecol. 1987;1(1):25-36.
73. Heusner AA. Size and power in mammals. J Exp Biol. 1991;160:25-54.
74. Vinogradov AE. Nucleotypic effect in Homeotherms: Body-mass-corrected basal metabolic rate of mammals is related to genome size. Evolution. 1995;49(6):1249-59.
75. Bromham L, Rambaut A, Harvey PH. Determinants of rate variation in mammalian DNA sequence evolution. J Mol Evol. 1996;43:610-21.
76. White CR, Phillips NF, Seymour RS. The scaling and temperature dependence of vertebrate metabolism. Biol Lett. 2006 Mar 22;2(1):125-7.
77. Jones KE, Bielby J, Cardillo M, Fritz SA, O'Dell J, Orme CD, et al. PanTHERIA: a species-level database of life history, ecology, and geography of extant and recently extinct mammals. Ecology. Ecol Lett. 2009;90: 2648.
78. Hayssen V, Lacy RC. Basal metabolic rates in mammals: Taxonomic differences in the allometry of BMR and body mass. Comp Biochem Physiol A Mol Integr Physiol. 1985;81(4):741-54.
79. Minetti AE, Ardigò LP, Reinach E, Saibene F. The relationship between mechanical work and energy expenditure of locomotion in horses. J Exp Biol. 1999;202:2329-38.
80. Lovegrove BG. The zoogeoghraphy of mammalian basal metabolic rate. Am Nat. 2000;156(2):201-19.
81. Lovegrove BG. The influence of climate on the basal metabolic rate of small mammals: a slow-fast metabolic continuum. J Comp Physiol B. 2003 Mar;173(2):87-112.
82. Jarsky TM. Effects of hypoxia and hypercapnia on circadian rhytms in the golden hamster (*Mesocricetus auratus*). J Appl Physiol. 2000;89:2130-8.
83. Clarke A, Rothery P, Isaac NJ. Scaling of basal metabolic rate with body mass and temperature in mammals. J Anim Ecol. 2010 May;79(3):610-9.
84. Metcheva R, Beltcheva M. Bioenergetic Characteristics of Genus *Mus* (Mammalia: Rodentia) from South Europe. Acta Zool Bulg. 2012;4:125-31.
85. Brooks DE. Nucleotides in spermatozoa. J Reprod Fertil. 1971;25: S302-S303.
86. Söderquist L. ATP content and sperm motility of extended bovine semen under different storage conditions. Acta Vet Scand. 1991;32: 511-508.
87. Kakar SS, Anand SR. Changes in adenosine 5'-triphosphate, adenilate energy charge and adenosine 3'5'-cyclyc monophosphate during the freezing of buffalo semen. J Reprod Fertil. 1981;62: 543-548.
88. Ford WC. Glycolysis and sperm motility: does a spoonful of sugar help the flagellum go round? Hum Reprod Update. 2006;12: 269-274.
89. Long JA, Guthrie HD. Validation of a rapid, large-scale assay to quantify ATP concentration in spermatozoa. Theriogenology. 2006;65: 1620-1630.
90. Guenzel-Apel AR, Ekrod B. Influence of seminal plasma and extender on sperm motility, ATP-concentration, and the activity of acid and alkaline phosphatases of beagle dog semen. Reprod Domest Anim. 1991;26: 31-41.
91. Minelli, A., Moroni M, Castellini C, Lattaioli P, Mezzasoma I, Ronquist G. Rabbit spermatozoa: a model for studying ATP homeostasis and motility. J Androl. 1999;20: 259-266.
92. Heiskanen M, Pirhonen A, Koskinen E, Maenpaa PH. Motility and ATP content of extended equine spermatozoa in different storage conditions. J Reprod Fertil. 1987;35: 103.
93. Ford WC, Harrison A, Takkar GL, Waites GM. Inhibition of glucose catabolism in rat, hamster, Rhesus monkey and human spermatozoa by α-Chlorohydrin. Int J Androl. 1979;2: 275-288.
94. Ford WC, Harrison A. Effect of α-chlorohydrin on glucose metabolism by spermatozoa from the cauda epididymidis of the rhesus monkey (*Macaca mulatta*). J Reprod Fertil. 1980;60: 59-64.
95. Stephens DT, Critchlow LM, Hoskins DD. Mechanism of inhibition by gossypol of glycolysis and motility of monkey spermatozoa in vitro. J Reprod Fertil. 1983;69: 447-452.
96. Vigue C, Vigue L, Huszar G. Adenosine triphosphate (ATP) concentrations and ATP/adenosine diphosphate ratios in human sperm of normospermic, oligospermic, and asthenospermic specimens and in thier swim-up fractions: lack of correlation between ATP parameters and sperm creatine kinase concentrations. J Androl. 1992;13: 305-311.
97. McLaughlin EA, Ford WC, Hull MGR. Adenosine triphosphate and motility characteristics of fresh and cryopreserved human spermatozoa. Int J Androl. 1994;17: 19-23.
98. Hyne RV, Edwards KP. Influence of 2-deoxy-D-glucose and energy substrates on guinea-pig sperm capacitation and acrosome reaction. J Reprod Fertil. 1985;73: 59-69.
99. Rogers BJ, Morton B. ATP levels in hamster spermatozoa during capacitation in vitro. Biol Reprod. 1973;9: 361-369.
100. Miki K, Qu W, Goulding EH, Willis WD, Bunch DO, Strader LF, et al. Glyceraldehyde 3-phosphate dehydrogenase-S, a sperm-specific glycolytic enzyme, is required for sperm motility and male fertility. Proc Natl Acad Sci U S A. 2004;101: 16501-16506.
101. Xia J, Reigada D, Mitchell CH, Ren D. CATSPER channel-mediated Ca2+ entry into mouse sperm triggers a tail-to-head propagation. Biol Reprod. 2007;77: 551-559.
102. Ford WC, Harrison A. The effect of 6-chloro-6-deoxysugars on adenine nucleotide concentrations in and motility of rat spermatozoa. J Reprod Fertil. 1981;63: 75-79.
103. Jeulin C, Soufir J. Reversible intracellular ATP changes in intact rat spermatozoa and effects on flagellar sperm movement. Cell Motil Cytoskel. 1992;21: 210-222.
