# Supplementary material for: Mass-Specific Metabolic Rate Influences Sperm Performance through Energy Production in Mammals
Source: PLoS One. 2015 Sep 15;10(9):e0138185. doi: 10.1371/journal.pone.0138185 (PMC4570794; doi:10.1371/journal.pone.0138185)
Supplement: S2 Table — Phylogenetically controlled multiple regression analyses (PGLS). Superscripts following the λ value indicate significance levels (n.s. p>0.05; *p<0.05) in likelihood ratio tests against models with λ = 0 (first position) and λ = 1 (second position). Effect size r calculated from the t values and the non-central 95% confidence limits (CLs) for the z-transformed value of r are presented. Confidence intervals excluding 0 indicate statistically significant relationships. P-values and CL that indicate statistical significance are shown in bold. All variables were log10 transformed. n: number of species. (PDF) [file pone.0138185.s004.pdf]

S2 Table. Relations among mass-specific metabolic rate, ATP amount, and relative testes size in eutherian mammals.

Dependent variable	Independent variable	Slope	R^2	*p*- value	*t* -value	λ value	Effect size	CL(-)	CL(+)	n
Mass-specific metabolic rate	Body mass	-0.3201	0.93	**<0.0001**	-8.8406	<0.001[ns,*]	-0.8462	**-1.5945**	**-0.8905**	34
	Testes mass	0.0744		0.1416	1.5082		0.2615	-0.0844	0.6197	
ATP amount per sperm	Body mass	-0.2928	0.43	**0.0495**	-2.0984	0.025[ns,*]	-0.4338	**-0.9142**	**-0.0149**	22
	Testes mass	0.2538		0.1928	1.3503		0.2959	-0.1446	0.7547	
Length-adjusted ATP concentration	Body mass	-0.1396	0.18	0.1600	-1.4624	<0.001[ns,*]	-0.3181	-0.7792	0.1202	22
	Testes mass	0.1424		0.2821	1.1069		0.2461	-0.1984	0.7009	

Phylogenetically controlled multiple regression analyses (PGLS). Superscripts following the λ value indicate significance levels (n.s. $p > 0.05$; $*p < 0.05$) in likelihood ratio tests against models with $\lambda = 0$ (first position) and $\lambda = 1$ (second position). Effect size *r* calculated from the *t* values and the non-central 95% confidence limits (CLs) for the *z*- transformed value of *r* are presented. Confidence intervals excluding 0 indicate statistically significant relationships. *P* -values and CL that indicate statistical significance are shown in bold. All variables were $\log_{10}$ transformed. n: number of species.
